# On the Treatment of Chilblains

**Published:** 1849-12

**Authors:** 


*On the Treatment of Chilblains.* By M. OSSIEUR.—In the earliest stage, friction, either employed dry, or with brandy or spirits of camphor, is the simplest and best means; but when the parts have become red, swollen, shining, and even covered with phlyctenæ, but prior to ulceration, the formula recommended by M. Goffin may be used with the greatest advantage:—Camphor, 4 parts; Ess. Oil Turpentine, 30 parts. When the practitioner is only consulted after ulceration has for some time taken place, M. Devergie's ointment is then the best application:—

Lard, 1 oz.;
Liq. Plumb. Subac., 12 drops;
Thebaic Extract, 3 grains;
Creosote, 10 drops.

*Bulletin de Therapeutique*, vol. xxxvi, p. 38.

